# MRP3: a molecular target for human glioblastoma multiforme immunotherapy. 

**DOI:** 10.1186/1471-2407-10-468

**Published:** 2010-09-01

**Authors:** Chien-Tsun Kuan, Kenji Wakiya, James E Herndon, Eric S Lipp, Charles N Pegram, Gregory J Riggins, Ahmed Rasheed, Scott E Szafranski, Roger E McLendon, Carol J Wikstrand, Darell D Bigner

**Affiliations:** 1 Department of Pathology, Duke University Medical Center, Durham, North Carolina 27710, USA; 2 Preston Robert Tisch Brain Tumor Center, Duke University Medical Center, Durham, North Carolina 27710, USA; 3 Department of Neurosurgery, Johns Hopkins University, Baltimore, Maryland 21210, USA; 4 Department of Microbiology, Saba University School of Medicine, Saba, Netherlands Antilles, USA

## Abstract

**Background:**

Glioblastoma multiforme (GBM) is refractory to conventional therapies. To overcome the problem of heterogeneity, more brain tumor markers are required for prognosis and targeted therapy. We have identified and validated a promising molecular therapeutic target that is expressed by GBM: human multidrug-resistance protein 3 (MRP3).

**Methods:**

We investigated MRP3 by genetic and immunohistochemical (IHC) analysis of human gliomas to determine the incidence, distribution, and localization of MRP3 antigens in GBM and their potential correlation with survival. To determine MRP3 mRNA transcript and protein expression levels, we performed quantitative RT-PCR, raising MRP3-specific antibodies, and IHC analysis with biopsies of newly diagnosed GBM patients. We used univariate and multivariate analyses to assess the correlation of RNA expression and IHC of MRP3 with patient survival, with and without adjustment for age, extent of resection, and KPS.

**Results:**

Real-time PCR results from 67 GBM biopsies indicated that 59/67 (88%) samples highly expressed *MRP3 *mRNA transcripts, in contrast with minimal expression in normal brain samples. Rabbit polyvalent and murine monoclonal antibodies generated against an extracellular span of MRP3 protein demonstrated reactivity with defined *MRP3*-expressing cell lines and GBM patient biopsies by Western blotting and FACS analyses, the latter establishing cell surface MRP3 protein expression. IHC evaluation of 46 GBM biopsy samples with anti-MRP3 IgG revealed MRP3 in a primarily membranous and cytoplasmic pattern in 42 (91%) of the 46 samples. Relative RNA expression was a strong predictor of survival for newly diagnosed GBM patients. Hazard of death for GBM patients with high levels of *MRP3 *RNA expression was 2.71 (95% CI: 1.54-4.80) times that of patients with low/moderate levels (p = 0.002).

**Conclusions:**

Human GBMs overexpress MRP3 at both mRNA and protein levels, and elevated MRP3 mRNA levels in GBM biopsy samples correlated with a higher risk of death. These data suggest that the tumor-associated antigen MRP3 has potential use for prognosis and as a target for malignant glioma immunotherapy.

## Background

Glioblastoma multiforme (GBM) is the most common and aggressive neuroectodermal neoplasm in adults. Although a recent study showed meaningful survival benefit associated with chemotherapy using a temozolomide-based chemoradiation approach [1], the median progression-free survival among patients treated with this regimen was only 7.9 months, and the overall survival was only 14.6 months [1]. Thus, an effective treatment for GBM patients is still a critical need.

Tumor-specific antigens that can be targeted by monoclonal antibodies (MAbs) conjugated with either radioisotopes or cytotoxins have great potential for cancer therapy [2,3]. MAbs have been applied to the treatment of malignant gliomas through selective destruction of tumor cells and sparing of normal brain cells [4,5]. Glioma-associated antigens targeted by immunotherapeutic approaches include cell adhesion molecules, matrix proteins, and growth factor receptors, such as tenascin [6], wild-type epidermal growth factor receptor [7], its glioma-associated variant, epidermal growth factor receptor variant III [5,8], and GPNMB [9]. Notwithstanding the tumor-restricted presentation of these antigens, GBMs are a heterogeneous group of tumors, consisting of genotypically and phenotypically divergent populations of cells [10,11]. As a result, antigenic expression profiles show a significant level of variation among and within individual GBMs [12]. Antigenic drift, observed in cultured cells, suggests the possibility of antigenic escape in primary brain tumors during treatment. Thus in any immunotherapeutic regimen, the antigenic heterogeneity seen in GBM necessitates the precise and timely selection of one or more target molecules for each patient. One approach to circumvent neoplastic cell heterogeneity is to expand the spectrum of GBM-specific targetable molecules and to customize therapy by using the best combination of targeted tumor antigens. Recent advances in genome technology have made it possible to analyze systematically the differences in gene expression patterns between normal and cancer cells, providing opportunities to discover novel antigens with tumor-specific distribution [13,14].

Multidrug-resistance protein 3 (MRP3), also known as the ATP-binding cassette (ABC) superfamily C Member 3, or ABCC3, is an organic anion transporter that we have recently identified as a candidate GBM marker by the serial analysis of gene expression (SAGE) method [15]. The best-studied mechanisms of multidrug resistance in malignant cells involve the overexpression of ATP-driven anticancer drug efflux pumps of the ABC superfamily [16]. MRP3 is involved in ATP-dependent transport of hydrophobic compounds [17] and of bile acids under certain physiological conditions [18]. MRP3 has limited distribution in human normal tissues and is expressed in adrenal gland, kidney, placenta, and organs of the gastrointestinal tract, including intestine, pancreas, liver, and gallbladder [19,20]. Recent work using *MRP3*-transfected cell lines has demonstrated the ability of MRP3 to transport certain classes of cytotoxic anticancer agents [21-23]. Accumulating evidence indicates that *MRP3 *gene expression is ectopically activated during carcinogenesis. *MRP3*, as mRNA or protein, has been detected in a variety of human cancer cell lines and tissues, including malignant gliomas [24-30], which implies possible involvement of MRP3 in the acquisition of a drug-resistant phenotype in these tumors.

Calatozzolo et al. showed that, in contrast to expression levels in nontumor brain samples, normal human astrocytes, and cultured endothelial cells, *MRP3 *is hyperexpressed in astrocytomas as the primary resistance to chemotherapy with drugs like cis-platinum (CDDP) and carmustine (BCNU) [31] and that MRP3 can modulate drug sensitivity to certain anticancer agents, such as cisplatin, vincristine, and etoposide, in human gliomas [25]. It has recently been shown that hepatic progenitor cells have high expression levels of functional MRP1 and MRP3, which may have a role in removing either exogenous or endogenous toxins and metabolites from progenitor cells [32]. Expression of ABC transporters was also found to be responsible for the highly enriched population phenotype in a wide variety of stem cells [33], and other initial studies also showed that they may be active in hematopoietic stem cells as functional regulators [34]. Although MRP3 was much less expressed in cancer stem cells before differentiation, after differentiation, the expression of MRP3 notably rose, which suggests that just after differentiation the cells acquired chemotherapeutic resistance via MRP3 [35].

In our preliminary survey of a limited number of glioma samples, *MRP3 *mRNA transcripts were highly expressed (80- to 100-fold induction) in comparison with normal brain tissues [15]. MRP3 could be an important target for immunotherapy, if the expression of *MRP3 *RNA by gliomas and minimum of expression in normal brain is reflective of actual MRP3 protein expression. This, along with its transmembrane localization, makes MRP3 an attractive target in immunotherapy for malignant gliomas. More pertinent, MRP3 is a tumor rejection antigen recognized by cytotoxic T lymphocytes in human lung adenocarcinomas [36]. However, little is known about *MRP3 *mRNA and protein expression in a large number of newly diagnosed GBM patient samples. We have therefore proposed to investigate the suitability of this *MRP3 *marker as a glioma immunotherapeutic target.

In this study, we conducted a genetic and immunohistochemical (IHC) evaluation of human gliomas to determine the incidence, distribution, and pattern of localization of MRP3 antigens in brain tumors. To assess the therapeutic potential of MRP3 as a GBM-associated antigen, we used real-time reverse transcription-polymerase chain reaction (RT-PCR) to determine *MRP3 *RNA transcript levels in 67 biopsy specimens from newly diagnosed GBM patients. In addition, a panel of 46 newly diagnosed GBM cases was assessed for MRP3 protein expression by IHC analysis, and survival analyses were performed based on these two parameters. We provide evidence that *MRP3 *mRNA expression is up-regulated in the majority of GBM cases and that glioma cells express MRP3 protein *in vitro *and *in vivo*. Increased *MRP3 *mRNA expression in GBM biopsy samples correlated with a higher risk of death. We demonstrate that MRP3 is a good potential molecular target for the immunotherapeutic treatment of malignant gliomas.

## Methods

### Cell lines

Human GBM-derived cell lines D54 MG, D247 MG, D392 MG, and D245 MG were established and maintained in our laboratory [10]; T98G and U251 MG were obtained from the ATCC. Glioma cells were grown in Zinc Option medium supplemented with 10% FCS (ZO-10% FCS). Cell lines with defined expression of MRP molecules were the kind gift of Dr. Piet Borst [21,37,38]. This defined cell line system consists of the nontransfected parent cell line 2008 (human ovarian carcinoma) transfected to express MRP1, MRP2, or MRP3. The HEK293 (human embryonic kidney) cell line was transfected to express MRP4. The MCDK (canine kidney) cell line was transfected to express MRP5. The monospecificity of MRP expression by this panel has been established [38]; all cell lines were grown in 10% FCS-DMEM (GIBCO/BRL 15050-022 [Invitrogen Corp., Carlsbad, CA]; with high glucose and phenol red) and split with 0.05% trypsin/EDTA.

For transient and stable expression, the cDNA of *MRP3 *was ligated into the mammalian expression vector pcDNA3.1 (Invitrogen, Carlsbad, CA). The resulting pcDNA3.1-*MRP3 *was introduced into HEK293 cells by using the Lipofectamine 2000 reagent (Invitrogen), and transiently transfected cells were harvested 48 h later. Stably transfected clones were selected in medium containing 200 μg/ml of Zeocin (Invitrogen).

### Brain tumor samples

Samples of primary GBM (Grade IV, according to WHO criteria [39]) tumors were obtained from 94 newly diagnosed patients at the Department of Neurosurgery, Duke University Medical Center. Among those 94 GBM samples, as summarized for the patient characteristics in Table 1, 67 cases were evaluated by quantitative RT-PCR analysis for MRP3 mRNA transcript expression and 46 cases were evaluated by IHC analysis for MRP3 protein expression. No patient had any history of chemotherapy or radiotherapy before surgery. The samples were immediately snap-frozen in liquid nitrogen and stored at -80°C until analysis.

**Table 1:** Patient Characteristics

Patient characteristic	All patients	Patients with MRP3 IHC Data	Patients with MRP3 mRNA Data
# of Patients	94	46	67
Gender:			
Male	59 (63%)	30 (65%)	39 (42%)
Female	35 (37%)	16 (35%)	28 (59%)
Age:			
≤ 45 years	22 (23%)	10 (22%)	13 (19%)
>45 years	72 (77%)	36 (78%)	54 (81%)
KPS:			
90-100	51 (54%)	23 (50%)	38 (57%)
70 - 80	36 (38%)	21 (46%)	24 (36%)
50 - 60	5 (5%)	2 (4%)	3 (4%)
Unknown	2 (2%)	0	2 (3%)
Extent of Resection:			
GTR	75 (80%)	34 (74%)	55 (82%)
STR	17 (18%)	10 (22%)	12 (18%)
Biopsy	2 (2%)	2 (4%)	0
Post-operative Treatment:			
CPT-11	2 (2%)	2 (4%)	1 (1%)
CPT-11/Celebrex	2 (2%)	2 (4%)	0
Cyclophosphamide/Thalidomide	1 (1%)	1 (2%)	0
Gliadel	4 (4%)	2 (4%)	2 (3%)
Gliadel→XRT	7 (7%)	5 (11%)	7 (10%)
Gliadel→XRT/Temo	4 (4%)	2 (4%)	2 (3%)
Mab I^131^-81C6	8 (9%)	5 (11%)	7 (10%)
Temozolomide	3 (3%)	2 (4%)	2 (3%)
Temozolomide/CPT-11	1 (1%)	0	1 (1%)
Temozolomide/Avastin	1 (1%)	0	1 (1%)
Vincristine/Cisplatin/Cyclophosphamide	1 (1%)	1 (2%)	0
XRT	19 (20%)	12 (26%)	15 (22%)
XRT/Temo	37 (39%)	11 (24%)	26 (39%)
None/Unknown	4 (4%)	1 (2%)	3 (4%)
Survival Status:			
Alive	7 (7%)	3 (7%)	5 (7%)
Dead	87 (93%)	43 (93%)	62 (93%)
Available Data:			
RNA only	48 (51%)	0	48 (72%)
IHC only	27 (29%)	27 (59%)	0
RNA and IHC	19 (20%)	19 (41%)	19 (28%)

Abbreviations: IHC: immunohistochemical, GTR: Gross Total Resection, STR: Subtotal Resection, XRT: External Beam Radiation Therapy.

### Quantitative RT-PCR assay

Total cellular RNA for use in the quantitative RT-PCR assay was isolated from confluent cultured cells and tumor tissues by using an RNeasy Mini Kit (Qiagen, Valencia, CA) and then treated with RNase-free DNase I (Ambion, Austin, TX). Total RNA of normal adult whole brain was either purchased from Clontech (Palo Alto, CA) or obtained from the Brain Tumor BioRepository, Preston Robert Tisch Brain Tumor Center, Duke University Medical Center. These total RNA samples (0.2 μg) were converted to random-primed cDNA with SuperScript II RNaseH^- ^Reverse Transcriptase (Life Technologies, Rockville, MD) in a reaction volume of 20 μl. After reverse transcription, 280 μl of water was added to the reaction mixture, and 2 μl of diluted cDNA sample was used as a template in PCR experiments.

The synthesized first-strand cDNA samples were subjected to real-time PCR with SYBR Green dye. Incorporation of SYBR Green dye into the PCR products was monitored with an ABI PRISM 7900HT Sequence Detector System (Applied Biosystems, Foster City, CA). For PCR reactions, 2 μl of cDNA, 5 μl of 2× SYBR Green PCR Master Mix (Applied Biosystems, Warrington, UK), and 200 nM of each primer were used in a total volume of 10 μl. Cycling parameters were 50°C for 2 min and 95°C for 10 min, followed by 40 cycles of 95°C for 15 s and 64°C for 30 s. For each sample, *β-actin *transcript was amplified simultaneously as an internal control. To avoid amplification from contaminating genomic DNA, primer pairs were designed to amplify sequences spanning introns. The oligonucleotide primer sequences were as follows.

*MRP3 *(sense) 5'-TGCCATCGACCTGGAGACTGACAAC-3'

*MRP3 *(antisense) 5'-GACATATTTGGTGTCATTTCCTTCCTGATG-3'

*β-actin *(sense) 5'-CCAACCGCGAGAAGATGACCCAGATCATGT-3'

*β-actin *(antisense) 5'-GGTGAGGATCTTCATGAGGTAGTCAGTCAGG-3'

The integrity of PCR products was confirmed by dissociation curve analysis using SDS 2.0 software (Applied Biosystems) and agarose gel electrophoresis followed by ethidium bromide staining. The threshold cycle (C_T_) values were determined for *MRP3 *and corresponding *β-actin *genes. To normalize the amount and quality of total RNA used in cDNA synthesis, the *MRP3/β-actin *ratio was calculated from the following formula for each sample:

$$MRP3/\beta -actin\text{ ratio}={\text{2}}^{\left({\text{C}}_{\text{T}}\beta -actin-{\text{C}}_{\text{T}}MRP3\right)}$$

In each tumor, relative *MRP3 *mRNA expression levels were measured in terms of fold induction ratio over normal whole-brain sample, which was determined by dividing the *MRP3/β-actin *ratio of the tumor sample by that of control normal whole brain. All measurements were performed in triplicate wells and repeated twice. Positive cases were defined as those with *MRP3 *RNA levels 3-fold higher than normal brain.

### MRP3 fusion protein

**Figure 1:**
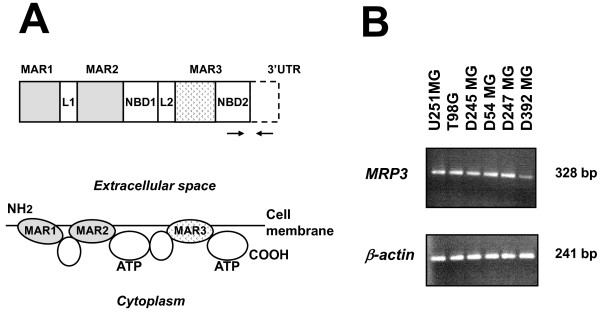
**Structure of MRP3 and expression of MRP3 mRNA in glioblastoma-derived cell lines**. (A) MRP3 structure. ***Upper***: the organization of MRP3. ***Lower***: membrane topology model for MRP3 (modified from Hipfner et al. [40]). MAR1, MAR2, MAR3, membrane-associated regions 1-3; L1, L2, linker domains 1 and 2; NBD1, NBD2, nucleotide-binding domains 1 and 2. Positions of PCR primers are indicated by arrows. (B) RT-PCR results showing *MRP3 *mRNA expression (***Upper***) and β-*actin *expression (***Lower***) in GBM cell lines U251 MG, T98G, D245 MG, D54 MG, D247 MG, and D392 MG.

To obtain antibodies reactive with the extracellular epitopes of MRP3, a fusion protein consisting of the *Escherichia coli *maltose-binding protein (MBP) and one of the membrane-associated regions (MARs) of MRP3 was constructed in the plasmid vector pMAL-c2X (New England BioLab, Beverly, MA). The *MRP3 *sequence in the expression vector encoded a 241-amino acid fragment (aa 984 to 1224) of MRP3 and was designated as MAR3 (Fig. 1A). The boundaries of MRP3 domains were determined according to the membrane topology model for MRP1 [40]. The DNA fragment encoding the MAR3 domain of MRP3 was PCR amplified from the plasmid vector pGEM7Zf-MRP3 containing full-length *MRP3 *cDNA (kindly provided by Dr. Marcel de Haas, The Netherlands Cancer Institute, Amsterdam) with the following primers.

sense: 5'-TCGGATCCGGCACCATGGCTGCCATTGGAGCCAATGTG-3'

antisense: 5'-AGTCTAGATTACCCCGGGTTCAGGCTGCTCCT-3'

After digestion with *Bam*HI and *Xba*I, the *MAR3 *DNA fragment was cloned into the corresponding restriction site of pMAL-c2X. The cDNA fragment for the MAR3 domain was also amplified by RT-PCR by using the same PCR primers and total RNA isolated from human glioma cell line T98G as a template. DNA sequencing revealed that nucleotide sequence of the *MAR3 *segment of *MRP3 *expressed in T98G was the same as that in pGEM7Zf-MRP3 and in previous reports [21,41] (data not shown). The resulting fusion protein MAR3-MBP was produced in *E. coli *TG1 and purified from cytosol medium with amylose resin affinity chromatography according to the manufacturer's instructions.

### Immunization

Rabbits were given a primary s.c. immunization with 250 μg of recombinant protein MAR3-MBP emulsified 1:1 in Freund's complete adjuvant and were subsequently boosted with the same dose of antigen emulsified 1:1 in Freund's incomplete adjuvant at 4-week intervals. Serum titers were determined by ELISA versus MAR3-MBP or MBP alone as target antigens and by live-cell-ELISA using MRP3-expressing D247 MG cells as targets [8]. Sera obtained 12 days following the sixth immunization were passed over a staphylococcal protein A column as previously described [42], and the rabbit immunoglobulin concentration was determined by capture ELISA [42].

Balb/c mice (NCI Animal Production Program, Frederick, MD) were similarly immunized with protein MAR3-MBP on days 171 and 191, following a four-dose immunization regimen with pcDNA3.1-MAR3 over days 1--87; the DNA-alone immunization protocol failed to produce titers sufficient to warrant fusion. Following MAR3-MBP protein boosts, 50% endpoint titers to target were in excess of 1/10,000, and spleens of two mice were fused. MAbs were initially identified for positivity with MAR3-MBP and absence of reactivity with MBP by ELISA; subsequently, they were further screened on cell lines that were determined, by mRNA analysis and reactivity with the polyvalent rabbit anti-MAR3-MBP serum, to be MRP3 positive (Western blot and indirect immunofluorescence analysis). Anti-MAR3 MAb 16A11, of the IgG_2a _isotype, was chosen because of its superior reactivity in indirect FACS analysis with MRP3-expressing D54 MG cells.

### Western blotting

Cells were lysed in NP-40 lysis buffer (50 mM Tris-Cl, pH 8.0/150 mM NaCl/1% NP-40/1 mM phenylmethylsulfonyl fluoride/0.045 mg/ml aprotinin). Aliquots of 10 μg of total protein were separated by SDS-PAGE and transferred onto polyvinylidene difluoride membranes. Membranes were incubated with 3% non-fat milk in PBS-0.1% Tween 20 to block nonspecific binding and were then probed overnight at 4°C with rabbit anti-MAR3 antiserum 1708 in PBS-0.1% Tween 20 containing 1% milk. The protein bands were detected by horseradish peroxidase (HRP)-conjugated anti-rabbit IgG secondary antibody coupled with SuperSignal West Pico Chemiluminescence Kit (Pierce, Rockford, IL).

### Indirect FACS analysis

Flow cytometric analysis for MRP3 expression was performed as described previously [43]. Test reagents and wash buffers were kept on ice to ensure the detection of cell surface molecules without allowing internalization to occur. Target cells were detached from culture flasks by incubation with 0.02% EDTA/PBS. Then, 1 × 10^6 ^cells/reaction mixture were maintained in 0.5% paraformaldehyde/PBS for 10 min at 4°C, washed, resuspended in 150 μl of PBS containing 10% FBS, and blocked for 20 min at 4°C. After two washes, the samples were reacted with rabbit anti-MAR3 1708 purified IgG, normal rabbit IgG, MAb 16A11, or murine IgG_2a _(all at 10 μg/ml in PBS) and rotated for 1 h at 4°C. After two additional washes, cells were incubated with species-appropriate FITC-labeled secondary antibody for 30 min at 4°C and then analyzed on a Becton Dickinson FACSort flow cytometer and cell sorter system (Becton Dickinson, San Jose, CA).

### Quantitative FACS analysis

We have previously published our techniques for QFACS analysis in the EGFRvIII MAb-antigen system [43]; briefly, FITC-MAb 16A11 is reacted with a cocktail of beads (Bangs Laboratories, Inc., Fishers IN) which bind no (B1) or graded amounts (20,000-200,000 [B2-B5]) of murine IgG molecules. Regression analysis of bead-binding capacity versus FL1 height is performed, generating a straight line formula, from which we calculate the number of molecules of FITC-MAb 16A11 bound to the target cells of identical aliquots, assuming 1:1 stoichiometry.

### Anti-MRP antibodies

All anti-MRP reagents were obtained from Kamiya Biomedical Company (Seattle, WA). Anti-MRP1 (MRPm6, IgG_1_), anti-MRP-2 (M_2_II-12, IgG_2a_), and anti-MRP3 (M_3_II-9; IgG_1_) are all murine monoclonal reagents directed against the cytoplasmic domain epitopes of their respective targets. Anti-MRP4 (M_4_I-10) and anti-MRP5 (M_5_I-1), from the same source, are rat IgG_2a _MAbs, also directed against a cytoplasmic domain epitope.

### Analysis of cells grown on chambered slides

Target cells were plated at densities determined in previous assays to yield a 70-80% confluent monolayer in either 24 or 48 h. Our IHC procedure using Lab-Tek Chamber Slides (Electron Microscopy Sciences, Hatfield, PA) has been published previously [43]; however Triton X treatment was omitted, as detection of nuclear staining was not an objective. Due to the cytoplasmic location of the epitopes detected by commercial reagents, all assays were performed in the presence of saponin, to allow penetration of reagents to the cytoplasm. Following fixation, blocking, and permeabilization, primary reagents (all at 12.5 μg/ml) were applied, and the assay continued as described, with the exception that the secondary reagent used was biotinylated horse anti-mouse IgG (Zymed, San Francisco, CA) or biotinylated goat anti-rat IgG (Caltag) as appropriate. Slides were counterstained with hematoxylin and read by two independent observers.

### Immunohistochemistry

Immunohistochemical staining of frozen sections derived from human glioma tumors was performed as described previously [43] using rabbit polyvalent anti-MAR3 antiserum 1708 purified IgG. Briefly, frozen tissues were sectioned (5-7 mm), mounted on positively charged slides, air-dried for 30 min, and fixed in cold acetone for 30 s. Nonspecific protein binding sites were blocked at room temperature with PBS containing 10% normal goat serum for 30 min, followed by incubation with primary antibodies at 10 μg/ml for 2 h. Positive reactions were detected by using biotinylated goat anti-rabbit IgG and HRP-conjugated streptavidin (Zymed). Bound peroxidase was developed with diaminobenzidine for 5 min. As a negative primary reagent control, irrelevant normal rabbit immunoglobulin (10 μg/ml) was used.

Slides were counterstained with hematoxylin, dehydrated, mounted, coded, and read by two investigators; staining patterns were scored as focal, multifocal, or diffuse, both in perivascular and parenchymal distribution, with intensity of stain described as weak, moderate, or strong.

### Statistical analyses

Cox's proportional hazards model was used to examine the effect of MRP3 mRNA expression (≤10-fold vs >10-fold) and IHC (zero vs positive) on survival, which was computed from the date of pathology sample acquisition to date of death or last contact. Patient characteristics such as gender, age, KPS, extent of resection, histology, post-operative treatment, and survival status are shown in Table 1. Analyses were conducted with and without adjustment for known prognostic factors such as age (≤ 45 years vs >45 years) [9], extent of resection (GTR vs STR/biopsy), and KPS (90-100 vs <90). Backwards elimination was used to identify a parsimonious multivariable model (SAS statistical analysis package, Cary, NC).

## Results

### Quantification of MRP3 mRNA in glioblastoma-derived cell lines and GBM samples

**Figure 2:**
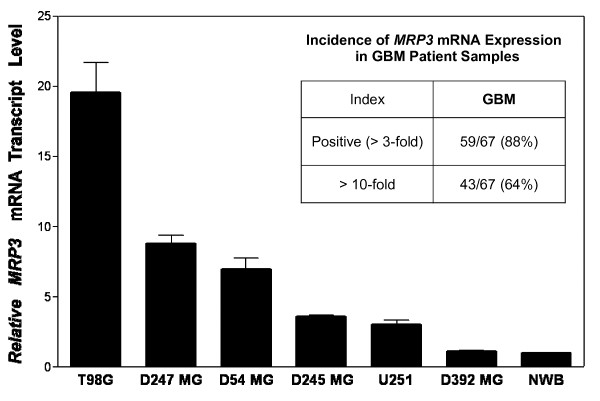
**Quantitative comparison of *MRP3 *mRNA levels in glioblastoma cell lines**. After normalization to β*-actin*, which had minimal variation in all normal and malignant samples that we tested, relative *MRP3 *mRNA transcript levels were expressed as compared to normal whole brain (NWB), which was set equal to 1. Results are expressed as the mean ± SD of four independent real-time PCR experiments, including a six-NWB sample average as the baseline standard. Inset: Incidence of *MRP3 *mRNA expression in GBM patient samples. Relative MRP3 mRNA expression was measured by quantitative RT-PCR in GBM tumor samples (n = 67) over NWB (n = 6). Results are the mean of triplicate measurements repeated twice. Positive cases were defined as those with *MRP3 *RNA levels 3-fold higher than normal brain; >10-fold: MRP3 RNA levels >10-fold those of normal brain.

We first assessed *MRP3 *mRNA expression levels in human GBM cell lines. All six GBM-derived cell lines examined expressed *MRP3 *mRNA transcripts at different levels (Fig. 1B and 1C). By quantitative RT-PCR, T98G, D247 MG, and D54 MG cells exhibited relatively high levels of *MRP3 *mRNA, ranging from 7.5- to 20-fold induction ratios over normal brain, whereas D245 MG and U251 MG exhibited moderate levels, a 3-fold induction ratio over normal brain, and the *MRP3 *mRNA level detected in D392 MG was similar to that in normal brain (Fig. 2). One leukemia cell line, HL60, was used as a negative control, and no RT-PCR products were detected (data not shown). Therefore, we used T98G, D247 MG, and D54 MG cells in analyses for MRP3 protein expression.

### Incidence of MRP3 mRNA expression in GBM patient samples

From the whole series of 94 newly diagnosed GBM patients, we measured *MRP3 *mRNA levels in 67 GBM biopsy samples. MRP3 mRNA expression was measured by quantitative RT-PCR in tumor samples and compared to that of normal whole brain (Fig. 2). Positive cases were defined as those with *MRP3 *RNA levels 3-fold higher than the level in normal brain. We found that 59 (88%) of the 67 GBM samples were positive, the *MRP3 *mRNA induction ranging from 3.5- to 370-fold over that in normal brain. Significantly, 43 (64%) of the 67 cases had expression of *MRP3 *mRNA greater than 10-fold over that seen in normal brain RNA extracts (data not shown). The expression levels of *MRP3 *transcripts were correlated positively with the grade level of gliomas (data not shown). Only minimal expression for the *MRP3 *gene was detected as a definable baseline in normal brain samples assayed simultaneously.

### Detection of MRP3 protein in glioblastoma cells

**Figure 3:**
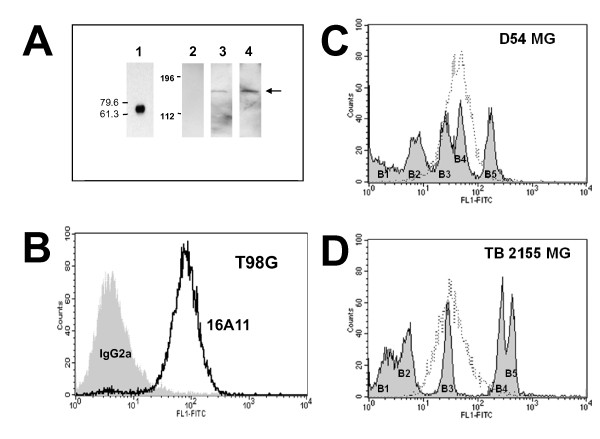
**Detection of MRP3 in a GBM cell line and in MRP3-transfected cells**. (A) Western Blot. Lane 1, reactivity of purified IgG from rabbit anti-MAR3 antiserum 1780 for fusion protein MAR3-MBP (~69 kD); lanes 2 and 3, protein extract (10 μg) of HEK293 cells transfected with the parental pcDNA3.1 and with pcDNA3.1-MRP3 plasmid, respectively; lane 4, protein extracts (10 μg) of *MRP3*-positive T98G cells that were electrophoresed and probed with rabbit anti-MAR3 antiserum 1708. The ~185-kDa mature MRP3 protein is indicated by an arrow. The locations of protein molecular weight markers are shown to the left. (B) Indirect FACS analysis. Reactivity of MAb 16A11 for non-permeabilized MRP3-expressing T98G cells, demonstration of a cell surface epitope. Irrelevant purified murine IgG_2a _was used as a negative control. (C) Quantitative FACS analysis of purified, directly fluoresceinated MRP3-specific MAb 16A11 with established human glioma cell line D54 MG; median MRP3 antigen density/cell: 4.4 × 10^4^. (D) Quantitative FACS analysis of MAb 16A11 with human glioma biopsy sample TB 2155 MG; median MRP3 antigen density/cell: 2.7 × 10^4^.

By Western blotting with rabbit polyclonal anti-MAR3 antiserum 1708, purified MAR3-MBP protein was detected as a 69-kDa protein band (Fig. 3A, lane 1). To establish the restricted specificity of this rabbit antiserum, Western blots were also performed using the HEK293 cells as a negative control (Fig. 3A, lane 2), the MRP3-transfected HEK293 cells, and the T98G glioblastoma cell line. MRP3 was detected in the MRP3-transfected HEK293 cells as well as the MRP3-positive T98G cells as an ~185-kDa protein (Fig. 3A, lanes 3 and 4), close to the molecular mass of MRP3 reported previously [21,23,44]. In indirect flow cytometric analyses under conditions measuring cell-surface-expressed targets, anti-MRP3 MAb 16A11 (Fig. 3B) and polyclonal antiserum 1708 reacted (data not shown) with *MRP3*-expressing T98G MG cells, demonstrating the existence of MRP3 epitopes on the cell surface membrane of live human GBM cells.

### Quantitative FACS analysis of MRP3 expression by cell lines, biopsies, and established xenografts with MAb 16A11

As described in Materials and Methods, MAb 16A11 was used to visualize the population profile of each sample, as well as to estimate the number of detected MRP3 molecules per cell surface, under conditions assuming 1:1 stoichiometry between antibody molecule and antigen, an assumption verified by comparison to standard Scatchard analysis with radioligand in the EGFRwt-EGF system [8]. The results of QFACS analysis of human-glioma-derived cell lines, biopsies, and xenografts are summarized in Table 2.

**Table 2:** Quantitative FACS analysis of MRP3 expression by biopsies, cell lines, and established xenografts with MAb 16A11

Diagnosis	Positive cases/Total cases	Percent	Estimated number of MRP3 molecules per cell
**Cell Lines**			
GBM adult	6/7	86	0.1-4.9 × 10^5 ^(med. = 1.9 × 10^5^)
MED pediatric	5/5	100	0.7--2 × 10^5 ^(med. = 1 × 10^5^)
**Biopsies**			
AA adult	3/6	50	1.7--2.7 × 10^4^
GBM adult	22/27	82	0.17--6.5 × 10^5 ^(med. = 6.5 × 10^4^)
**Xenografts**			
GBM adult	4/7	57	0.2--2.1 × 10^5^
MED pediatric	2/3	67	1.6--2.5 × 10^4^

We used FITC-MAb 16A11 with QFACS analysis to investigate MRP3 density on the cell surface of the cultured glioma cell lines shown to express MRP3 mRNA--D54 MG, D247 MG, T98G, U251, and D392 MG--and on human GBM biopsy samples. D54 MG cells were relatively homogeneous in expression of MRP3 on the cell surface (Fig. 3C), with a median density of 4.4 × 10^4 ^molecules per cell (6 repeat assays). D247 MG similarly expressed a median density of 2.3 × 10^4 ^molecules per cell, T98G expressed 0.38--4.9 × 10^5 ^molecules of MRP3 per cell (8 repeat assays; data not shown), U251 MG cells exhibited 0.4--2.1 × 10^5 ^MRP3 molecules per cell (3 repeat assays; data not shown), and D392 MG was uniformly negative for cell surface expression of MRP3 (4 repeat assays; data not shown).

Biopsies and xenografts of GBM tended to express lower cell surface MRP3 densities (median values in the 10^4 ^range) than did cell lines (median = 1.9 × 10^5^). Of six adult anaplastic astrocytoma (AA) biopsies analyzed (single assay in triplicate on freshly isolated cells), three were positive for cell surface MRP3, with a range in expression of 1.7--2.7 × 10^4 ^MRP3 molecules per cell (Table 2). Significantly, 22/27 adult GBM biopsies (82%) were positive for cell surface MRP3, with a range in expression of 0.17--6.5 × 10^5 ^(median = 6.5 × 10^4^) MRP3 molecules per cell (Table 2). Representative data from TB 2155 MG is shown in Fig. 3D (density/cell: 2.7 × 10^4^); again, the density distribution by these biopsy-derived cells is relatively homogeneous. This tendency was also observed in the medulloblastoma cell lines and xenografts examined. Of the six adult AA biopsy samples that were available for analysis, only 50% were positive, as opposed to 82% for GBM biopsies. Unlike the EGFRvIII variant, MRP3 appears to be maintained over time in cell culture. The slightly lower frequency of expression in established GBM xenografts (57%) may be preliminary due to the small sample size (7 cases).

### Immunohistochemical analysis of defined cell lines variously transfected to express MRP1-5

To validate the specificity of the polyvalent antiserum 1708 and MAb 16A11, we obtained the defined cell line system reported previously [21,37,38], which consists of the non-transfected parent cell line 2008 (human ovarian carcinoma) transfected to express either MRP1, MRP2, or MRP3, the HEK293 cell line transfected to express MRP4, and the MCDK canine kidney cell line transfected to express MRP5. The monospecificity of MRP expression by this panel has been established [38]. Defined antisera to each of the MRP proteins were used for comparison to the spectrum of reactivity exhibited by the immune reagents reported here, polyvalent rabbit antiserum 1708 and MAb 16A11. Untransfected parent cells were unreactive with any of the antibodies (Table 3, Parent column); shown here is the murine IgG_2a _irrelevant primary antibody control; normal rabbit IgG, controlled for concentrations of reference antisera, was also negative. The results demonstrate the specificity of the commercial reagents employed for their respective targets, and the single, appropriate MRP molecule expression by each of the transfectants (MRP1-tf, MRP2-tf, MRP3-tf, MRP4-tf, and MRP5-tf). Most importantly, MAb 16A11 and polyvalent serum 1708 are identical in their reactivity profile to the commercially available and consensus polyvalent antibody control for MRP3 detection, M_3_II-9: These three reagents detect only the MRP3-tf cell line.

**Table 3:** Immunohistochemical analysis of defined MRP-expressing cell lines*

Antibody	Parent	MRP1-tf	MRP2-tf	MRP3-tf	MRP4-tf	MRP5-tf
Negative Control	0	0	0	0	0	0
Anti-MRP1	0	2+	0	0	0	0
Anti-MRP2	0	0	2+	0	0	0
Anti-MRP3 (M3II-9)	0	0	0	2+	0	0
Anti-MRP3 (16A11)	0	0	0	2+	0	0
Anti-MRP3 (1708)	0	0	0	2+	0	0
Anti-MRP4	0	0	0	0	2+	0
Anti-MRP5	0	0	0	0	0	2+

*Parent, untransfected lines and their single MRP-transfected, derived lines (-tf) were grown on Lab-Tek chambers for IHC analysis. Primary antibodies and irrelevant controls were used at a concentration of 12.5 μg/ml (MAbs) or 10 μg/ml (polyvalent serum 1708 or normal rabbit IgG). Columns are headed by the target cell line designation; rows are labeled to the left with the primary antibody used for specific MRP detection. 0, 2+: levels of reactivity of the antibodies with each defined MRP-expressing cell line.

### Immunohistochemical analysis

We next investigated whether MRP3 proteins are expressed in human malignant glioma tissues. As described in Materials and Methods, polyvalent anti-MRP3 antiserum 1708 was used to detect the presence of MRP3 in frozen sections of GBM biopsy material. The results of this analysis are reported in Table 4. Immunostaining with rabbit anti-MRP3 IgG in 46 cases of GBM revealed MRP3, in a primarily membranous and cytoplasmic pattern, in 42 of the 46 GBM samples. Focal perivascular enhanced localization was seen in 18 of the 46 GBM samples. Normal CNS histology was also done with anti-MRP3 rabbit serum 1708 or MAbs such as 16A11, and no IHC staining was observed (data not shown).

**Table 4:** Summary of immunohistochemical evaluation of frozen GBM tissue sections with polyvalent anti-MRP3 antiserum 1708

Reactivity patterns	GBM
Parenchymal	
	22/46
	(48%)
Perivascular	
	2/46
	(4%)
Parenchymal and perivascular	
	18/46
	(39%)
Total reactive	
	42/46
	(91%)

**Figure 4:**
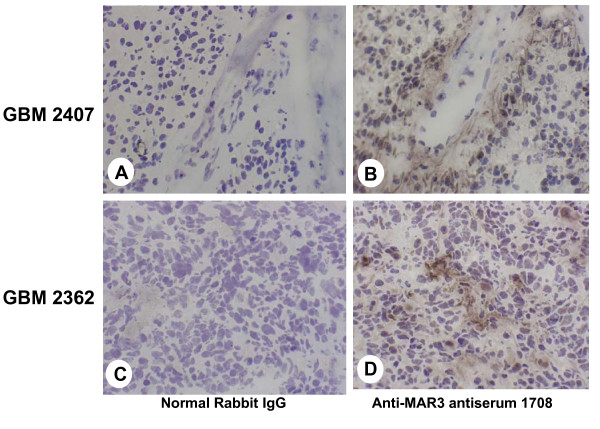
**Immunohistochemical analysis of frozen sections of human GBM with anti-MRP3 rabbit antiserum 1708**. (A and B) GBM 2407; (C and D) GBM 2362; all at 100×. (A and C) Normal rabbit IgG primary reagent control at an IgG concentration identical to that used for anti-MAR3 antiserum 1708 (B and D). (B) GBM 2407 exhibits multifocal staining by antibody 1708, with enhanced perivascular localization. (D) GBM 2362 provides an example of prominent focal cytoplasmic detection. Normal CNS histology was also stained with 1708, and no IHC reactivity was observed (results not shown).

The majority of cases (40/46 GBMs [87%], Table 4) exhibited prominent parenchymal staining (Fig. 4B and 4D), frequently coupled with multifocally enhanced perivascular localization (Fig. 4B). Even when the localization was focal in distribution, both cytoplasmic staining and membrane staining were seen (Fig. 4D). Control sections (Fig. 4A and 4C) probed with irrelevant normal rabbit immunoglobulin showed no reactivity. In general, the pattern of MRP3 immunoreactivity is predominantly perivascular and involves small veins more commonly than small arterioles. Tumor cell localization largely follows a perivascular enhancement (Fig. 4B). Rarely in astrocytic tumors (AA [result not shown] and GBM) is the entire biopsy sample reactive; the predominant pattern is multifocal or focal, with groups of reactive cells varying from weakly membranous to strongly cytoplasmic, and membranous localization adjacent to nonreactive cells (Fig. 4B and 4C). In contrast, oligodendrogliomas revealed a perinuclear-to-nuclear pattern in a heterogeneous mixture of reactive and nonreactive cells (result not shown).

### Statistical analyses

**Figure 5:**
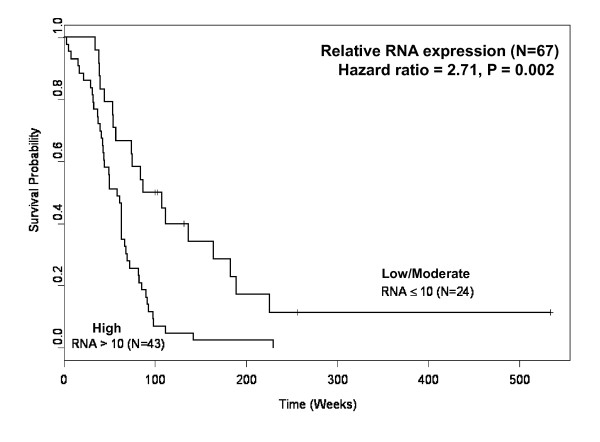
**Kaplan-Meier curve of time to progression stratified by MRP3 RNA transcript levels for newly diagnosed GBM patients**. Low/Moderate (n = 24): relative RNA expression levels less than or equal to 10-fold those of normal brain sample. High (n = 43): relative RNA expression levels greater than 10-fold those of normal brain sample. 95% Confidence interval: 1.54, 4.80; p = 0.002.

The characteristics of the 94 patients included in these analyses are summarized in Table 1, with a separate characterization of patients with IHC and RNA data. Overall, 87 of the 94 patients (93%) have died. The RNA expression data is from 67 newly diagnosed GBM patients and the IHC information from 46 newly diagnosed patients. We conducted survival analyses in a univariate model to examine the effects on survival of selected predictors such as age, KPS, extent of resection, RNA level of MRP3, and anti-MRP3 IHC positivity in specific patient subgroups and determined the hazard ratio (HR) associated with each predictor. These univariate analyses show the following: (1) Patients with a gross total resection live longer than patients with less than a gross resection (p = 0.0004; HR = 0.38 with 95% confidence interval 0.22, 0.64). (2) The mRNA level of MRP3 is a strong predictor of survival, with statistical significance (p = 0.002) in that patients with RNA levels less than or equal to 10-fold over normal brain level were predicted to live longer than patients with higher RNA levels (p = 0.002; HR = 2.71 with 95% confidence interval 1.54, 4.8), as shown in Fig. 5. However, (3) there was no statistically significant relationship between IHC reactivity and survival, and thus IHC positivity is not predictive of survival (p = 0.466).

Multivariable survival models were also run for survival analyses. Backwards elimination was used to generate these models while retaining either the IHC or the mRNA predictor in the model. In these multivariable analyses, IHC positivity is not predictive of survival (p = 0.723). However, after adjustment for extent of resection, high levels of RNA remain a significant predictor of survival (p = 0.001; HR = 2.54 with 95% confidence interval 1.44, 4.49).

## Discussion

In an ideal situation, target antigens for passive immunotherapy should be (1) tumor specific, that is, expressed in tumor and not normal tissue and (2) accessible (cell surface or matrix) at a density sufficient for targeting and significant cell kill. The criterion of absolute tumor specificity is rarely met, and current approaches often must target tumor-associated antigens (ratio of neoplastic tissue to normal tissue expression acceptable for lack of bystander effect).

In the present study, we have demonstrated that the *MRP3 *gene is expressed both at the mRNA level and the protein level in high-grade gliomas, but minimally in normal brain tissue. By quantitative RT-PCR analysis of 67 newly diagnosed GBM patient samples for *MRP3 *mRNA expression, 88% of the samples were positive, and 64% showed levels 10 times greater than the level of *MRP3 *mRNA expression in normal brain. Immunohistochemical analysis of 46 cases revealed that 91% of the GBM samples expressed MRP3 protein detectable by immunohistochemistry. The predominant staining pattern in all GBM was either parenchymal distribution only (22/46, 48%) or mixed perivascular and parenchymal in orientation, either focal or diffuse in distribution (18/46, 39%), with few cases showing only prominent perivascular distribution (2/46, 4%). As for the cellular origin of MRP3 expression in the bulk tumors, five of six GBM-derived cell lines examined in this study were positive for *MRP3 *mRNA transcripts (Fig. 2), this result supporting the view that MRP3 is produced mainly by transformed cells in glioma tissues.

Our results and those of other researchers indicate that *MRP3 *is highly expressed at gene and protein levels in human glioma cell lines and in clinical glioma specimens of grades III and IV [25]; consistent with other studies, we observed no apparent expression of *MRP3 *in normal brain tissue [45]. In addition, of the MRP family genes, *MRP1 *was also found to have increased mRNA expression in some human glioma cell lines and clinical samples [24,25]. However, MRP1 is known to be expressed in normal brain tissue [27]. *MRP4 *and *MRP5 *have been reported to exhibit expression in two glioblastoma cell lines, whereas *MRP2 *was not expressed in malignant glioma cells [24,25]. Bronger et al. have also detected the expression of other MRP family members, such as MRP4 and MRP5, in the blood-brain barrier and in the glioma cells at protein level [46]. Localization of MRP1, MRP4, and MRP5 protein in rapidly frozen perilesional samples of several regions of adult human brain was reported by Nies et al. [47]. Among normal vital organs outside the CNS, MRP3 protein expression has been observed in adrenal gland, kidney, and tissues associated with the circulation of bile acids [20].

However, the expression of MRP3 in normal tissues outside the CNS will not compromise the compartmental delivery of MRP3-related immunological agents within the CNS, because the optimal route for the administration of MAb-based therapeutic agents for tumors localized within the CNS is through surgically created resection cavities or saturation of an entire hemisphere by intracranial microdiffusion, called convection-enhanced delivery, to the brain tumor [48], which allows direct parenchymal infusion of therapeutics, bypassing the blood-brain barrier. Only trace amounts of therapeutics distribute systemically, and the possibility of life-threatening side effects, such as lung edema, would be minimal. However, these focal deliveries have limitations: Passive microdiffusion happens to only a very poor extent, given the gliosis reaction in the wall of the surgical cavity, and convection-enhanced delivery must take into account modern calculations for isodose delivery distributions to cover the relevant areas [49].

Localization to the tumor cell surface membrane or extracellular matrix is another prerequisite for target antigens in MAb-mediated therapy [50]. Amino acid sequence analysis has shown that in a topological model, MRP3 comprises seven domains, three of which contain multiple extracellularly expressed regions, while the remaining four are intracellularly located [40]. Although MRP3 protein is predicted to integrate in the cell surface membrane, the precise membrane topology of MRP3 is complex and remains unclear. Depending upon the software used, 16-18 transmembrane domains are predicted for MRP3, which complicates finding the precise location of extracellular epitopes of MRP3 [40]. To address this, our immunization was performed with fusion protein MAR3-MBP (MAR3; amino acids 984-1224 of MRP3), which may contain extracellular epitopes of MRP3, and serum titer was monitored by the reactivity with *MRP3*-expressing GBM cells under conditions detecting cell surface reactivity only. The resulting rabbit antiserum 1708 and murine MAb 16A11, derived from immunization with MAR3-MBP, recognized cell-surface-expressed MRP3 as defined by immunofluorescence analysis of *MRP3*-positive GBM cells under non-fixed conditions. These results from flow cytometry, along with IHC findings showing membranous staining of tumor cells, indicate the expression of MRP3 protein on the surface membrane of glioblastoma cells *in vitro *and *in vivo*. Recently, we have isolated scFvs M25 and M58, which specifically react with the extracellular N-terminus of MRP3, for IHC evaluation of human gliomas to determine the localization of MRP3 antigen. These Fv-based recombinant antibodies, possessing superior tumor penetration capabilities and selectively targeting tumor cells that express MRP3, may potentially be used in immunotherapy and diagnosis for brain tumors and other cancers [51].

Other factors that define the targetability of antigen molecules involve antigen density [52] and stability (lack of antigen shedding and internalization following binding to ligand or antibody) [50]. Results from immunotherapeutic experiments using xenograft models have revealed that the extent of tumor reduction elicited by a single MAb is generally proportional to the antigen density at the cell surface [52]. Although the minimum antigen density required for successful MAb-mediated therapy varies, depending on the mechanisms of action of cytotoxic conjugates, the general consensus is that ≥1 × 10^4 ^protein molecules per cell would be required for biologically effective targeting [43]. Preliminary data obtained by quantitative FACS analysis with MAb 16A11 directed against MAR3-MBP indicates that human malignant glioma cell lines T98G, D247 MG, and D54 MG express 0.3-4.9 × 10^5 ^MRP3 molecules per cell. More significantly, biopsy-derived GBM cells from 22/27 patients expressed MRP3 at a density ranging from 1.7 × 10^4 ^to 6.5 × 10^5 ^molecules per cell, well within the density range required for targeting.

The molecular mechanisms underlying overexpression of the *MRP3 *gene in gliomas remain to be determined. Activation of the MRP family genes can be achieved in several ways. For MRP1, an increase in *MRP1 *mRNA in cultured cell lines that have been selected for *in vitro *drug resistance is frequently associated with amplification of its cognate gene and at least some level of rearrangement [53], while elevated *MRP1 *mRNA found in human tumor samples occurs without [54] or only rarely with gene amplification [55]. Thus, further investigation could determine whether *MRP3 *mRNA overexpression in human gliomas also is associated with an increase of gene copy number. Up-regulation of *MRP3 *mRNA can occur by mechanisms other than gene amplification. Sequence analysis of the 5'-flanking region of the *MRP3 *gene has revealed that the *MRP3 *promoter lacks the typical TATA box and contains multiple potential binding sites for a number of transcription factors, including AP1, AP2, N-MYC, Sp1, and PEA-3 [41,56]. Alternatively, methylation of the promoter region, the stability of mRNA, or translational efficiency may be altered, which can contribute to the hyperexpression of MRP3.

The biological significance of aberrant expression of MRP3 in glioma tissues is not yet known. Our results and reports from other researchers [24,25] have demonstrated that *MRP3 *mRNA levels increase with tumor grade, indicating that MRP3 is likely to be a potential progression marker of glial tumors. In human colorectal carcinoma cell lines, the enhanced expression of *MRP3 *mRNA is associated with the inactivation of the *p53 *gene [57]. *MRP3 *gene expression may be regulated at the genetic level, depending on the status of oncogenes and/or tumor suppressor genes involved in the tumorigenesis of gliomas. On the other hand, one of the outstanding features of MRP3 is its inducibility by external stimuli, such as chemical carcinogens and anticancer drugs. This inducible nature of MRP3 has been observed in primate and rodent tissues and in a variety of cultured cells [58,59], and drug-induced MRP3 may provide an important mechanism for cytoprotection from the toxicity of xenobiotics. However, glioma cases examined in this study exhibited MRP3 overexpression *prior to *the exposure to antineoplastic reagents. MRP3 can also be up-regulated in response to a change in the intracellular redox state [57] and, as postulated for MRP1 [60], may improve cellular defense status against oxidative metabolites that accumulate during tumor growth.

The categorical variables of age, mRNA transcript level, and IHC staining intensity were used to construct Kaplan-Meier curves, and our univariate analysis demonstrated efficacy in predicting survival in the newly diagnosed GBM population. The univariate analyses show that patients with high *MRP3 *mRNA expression level had a higher risk (2.71 times higher) of death than the patients with low/moderate *MRP3 *mRNA expression level. The results of survival analysis suggest that the relative *MRP3 *mRNA levels represent a potential prognostic predictor of poor GBM patient survival. There are only a few molecular markers that are prognostic for survival in malignant gliomas. Elevated expression of *MRP3 *in GBM patient specimens, strong GST-π protein expression in human gliomas [61], a functional polymorphism in *EGF *[62], and high GPNMB expression in GBM patients [9] are associated with clinically more aggressive gliomas and are useful and powerful prognostic markers of poor patient survival.

## Conclusions

In conclusion, human GBMs overexpress *MRP3 *at both mRNA and protein levels, and elevated *MRP3 *mRNA levels in GBM biopsy samples correlated with a higher risk of death. These data indicate that the tumor-associated antigen MRP3 has potential use as a prognostic predictor for malignant gliomas. We further demonstrate that immunotherapeutic strategies designed to target MRP3 may be successful for treating malignant glioma patients.

## Abbreviations

AA: anaplastic astrocytoma; GBM: glioblastoma multiforme; KPS: Karnofsky performance score; FACS: fluorescence-activated cell sorter (analysis); HRP: horseradish peroxidase; IHC: immunohistochemical; MAb: monoclonal antibody; MAR: membrane-associated region; MBP: maltose-binding protein; MRP3: multidrug resistance protein 3; RT-PCR: reverse-transcription polymerase chain reaction; SAGE: serial analysis of gene expression

## Competing interests

The authors declare that they have no competing interests.

## Authors' contributions

CK designed, directed, and coordinated the study and drafted and finalized the manuscript. KW participated in the study design, performed all the molecular genetic and biochemical studies, and drafted part of the manuscript. JH participated in the design of the survival study and performed the statistical analysis. EL participated in the medical records retrieval for the survival study. GR participated in the study and helped to revise the manuscript. CP raised the rabbit polyclonal antibodies and carried out the immunoassays. AR and SS carried out the real-time PCR and immunoassays. RM contributed the critical interpretation of the IHC results. CW participated in the design of the study, raised murine monoclonal antibodies, performed the IHC and flow cytometric analyses, and helped to revise the manuscript. DB conceived of the study, participated in its design, and helped to revise the manuscript. All authors read and approved the final manuscript.

## Pre-publication history

The pre-publication history for this paper can be accessed here:

http://www.biomedcentral.com/1471-2407/10/468/prepub
